# The improvement effect of apple cider vinegar as a functional food on anthropometric indices, blood glucose and lipid profile in diabetic patients: a randomized controlled clinical trial

**DOI:** 10.3389/fcdhc.2023.1288786

**Published:** 2023-11-13

**Authors:** Sima Jafarirad, Mohammad-Reza Elahi, Anahita Mansoori, Abdollah Khanzadeh, Mohammad-Hossein Haghighizadeh

**Affiliations:** ^1^ Nutrition and Metabolic Diseases Research Center, Clinical Research Institute, Ahvaz Jundishapur University of Medical Sciences, Ahvaz, Iran; ^2^ Department of Nutrition, School of Allied Medical Sciences, Ahvaz Jundishapur University of Medical Sciences, Ahvaz, Iran; ^3^ School of Medicine, Abadan Faculty of Medical Sciences, Abadan, Iran; ^4^ Department of Epidemiology and Biostatistics, Ahvaz Jundishapur University of Medical Science, Ahvaz, Iran

**Keywords:** vinegar, blood glucose, insulin, cholesterol, low-density lipoprotein, high-density lipoprotein

## Abstract

**Background:**

Numerous medical costs are spent each year on treating and preventing the progression of diabetes. The positive effect of apple cider vinegar (ACV) has been shown on post-prandial hyperglycemia. This study aimed to evaluate the effects of prolonged consumption of ACV on blood glucose indices and lipid profile in patients with type 2 diabetes.

**Methods:**

This study was a randomized clinical trial and the participants were adults with type 2 diabetes. Participants were divided into two groups: ACV and control. The ACV group was treated with 30 ml of ACV per day. Both the intervention and control groups received the same recommendation for a healthy diet. Before and after eight weeks, fasting blood glucose, insulin, hemoglobin A1C, insulin resistance, total cholesterol (Chol), low-density lipoprotein (LDL), high-density lipoprotein (HDL) and triglyceride were measured.

**Results:**

Fasting blood glucose decreased after intervention in both groups, which was only significant in the ACV group (p = 0.01). There was a significant difference in hemoglobin A1C levels between the two groups (p < 0.001) after eight weeks. LDL was decreased in the ACV group (p < 0.001). Total Chol, LDL/HDL and Chol/HDL ratio decreased after the intervention period in the ACV group compared to the control group (p = 0.003, p = 0.001 and p = 0.001, respectively).

**Conclusion:**

Daily consumption of ACV may have beneficial effects in controlling blood glucose indices and lipid profile in patients with type 2 diabetes.

**Clinical trial registration:**

http://www.irct.ir, identifier IRCT20140107016123N13.

## 1 Introduction

Diabetes is one of the common chronic diseases and is associated with high blood glucose levels. The prevalence of diabetes is increasing worldwide (1). A global study reported that the number of people with diabetes has quadrupled in the last three decades, and diabetes is the ninth leading cause of death (2). In Iran, about 15% of adults suffer from diabetes (3). Diabetes can cause several complications, including retinopathy, nephropathy, high blood pressure, and cardiovascular disease (4). The risk of developing cardiovascular diseases (CVDs) in diabetic patients is 2 to 4 times that of non-diabetics (5). Patients with type 2 diabetes (T2D) account for 85% of all diabetic patients. Controlling of T2D includes the management of blood sugar, oxidative stress and the management of risk factors for CVDs (6).

Modern therapies have made significant advances in the control of T2D. However, given the social and financial consequences of diabetes and its effects on the healthcare system, researchers are interested in finding complementary and alternative therapies for managing and controlling diabetes and its complications (7).

Using alternative therapies, such as consumption of functional foods, is one way to control diabetes and its complications. The tendency of using natural supplements or foods to treat chronic diseases has increased significantly during the recent years (8). One of the oldest fermented food products is apple cider vinegar (9). Apple cider vinegar (ACV) is a plant-based food product made from apples at home or industrially. Its main component is acetic acid. In addition, it contains pectin, potassium, sodium, phosphorus, calcium, iron, ascorbic acid, thiamine, riboflavin, pyridoxine, biotin, folic acid, niacin, pantothenic acid and a variety of polyphenol compounds such as catechin, epicatechin, gallic acid, caffeic acid, syringic acid, p-coumaric acid, ferulic acid and chlorogenic acid (10, 11). ACV is used in many Iranian foods and as a home remedy (12) and could be accounted for as a functional food. Although the usage of ACV has a long history, the knowledge about its composition and therapeutic effects has recently received much attention (13). Some animal studies have shown the effect of ACV on blood glucose and lipid profile, which are two main goals in controlling diabetes (14, 15). To the best of our knowledge, few clinical trials have been conducted in this field. Most of them have examined the short-term postprandial effects of ACV on lipid profile and glucose levels, and they have reported contradictory results (16–19). It seems long-term clinical trials are necessary in this field. Therefore, this study aimed to evaluate the long-term effects of apple cider vinegar consumption on blood glucose, insulin, lipid profile, anthropometric indices and blood pressure in patients with T2D.

## 2 Methods

### 2.1 Ethics approval and consent of participate

This study was conducted according to the guidelines laid down in the Declaration of Helsinki. The Ethics Committee of Ahvaz Jundishapur University of Medical Sciences approved all procedures involving human subjects (ID: IR.AJUMS.REC.1397.476). All subjects provided written and informed consent and they were reassured that their information would be kept confidential. Informed consent was obtained from the legal guardian(s) or next of kin(s) of a few illiterate participants.

### 2.2 Participants

All participants were recruited from adult patients with T2D referred to the diabetes clinic of Ayatollah-Taleghani Hospital in Abadan (Khuzestan province, Iran). The sample size was calculated with 80% power and 95% confidence interval using the following formula.


$$n=\frac{{\left(Z_{1-\alpha_{/2}}+Z_{1-\beta }\right)}^{2}\left(S_{1}^{2}+S_{2}^{2}\right)}{{\left(\mu_{1}-\mu_{2}\right)}^{2}}$$

The sample size was determined based on a previous study regarding the changes in high-density lipoprotein-cholesterol concentration after consumption of ACV (20). Thirty-six subjects were determined in each two groups (a total of 72 subjects). We considered a 10% attrition rate so the final sample size was determined 80 subjects.

### 2.3 Study design

This study was a parallel-randomized, controlled, and open-labelled clinical trial. There were two arms in the study: the treatment (apple cider vinegar) and the control. The study was registered in the Iranian Clinical Trial Registration Center at http://www.irct.ir, (ID: IRCT20140107016123N13).

The inclusion criteria were diabetic type 2 patients. Based on the American Diabetes Association guidelines, one of the following criteria was considered as T2D: fasting plasma glucose ≥ 126 mg/dl, or oral glucose tolerance test ≥ 200 mg/dl (21). The other inclusion criteria were no oral supplement consumption, no daily vinegar intake in the last three months, no insulin injection, body mass index (BMI) between 18.5 kg/m^2^ and 34.9 kg/m^2^, age range 30 to 60 years, willingness to participate in the study, controlling the disease with diet and taking blood glucose lowering medicines or just taking blood glucose lowering medicines. Exclusion criteria were lack of proper cooperation, BMI more than 35, pregnancy and lactation, insulin injection, and gastrointestinal disorders including reflux and gastric ulcer.

**Figure 1:**
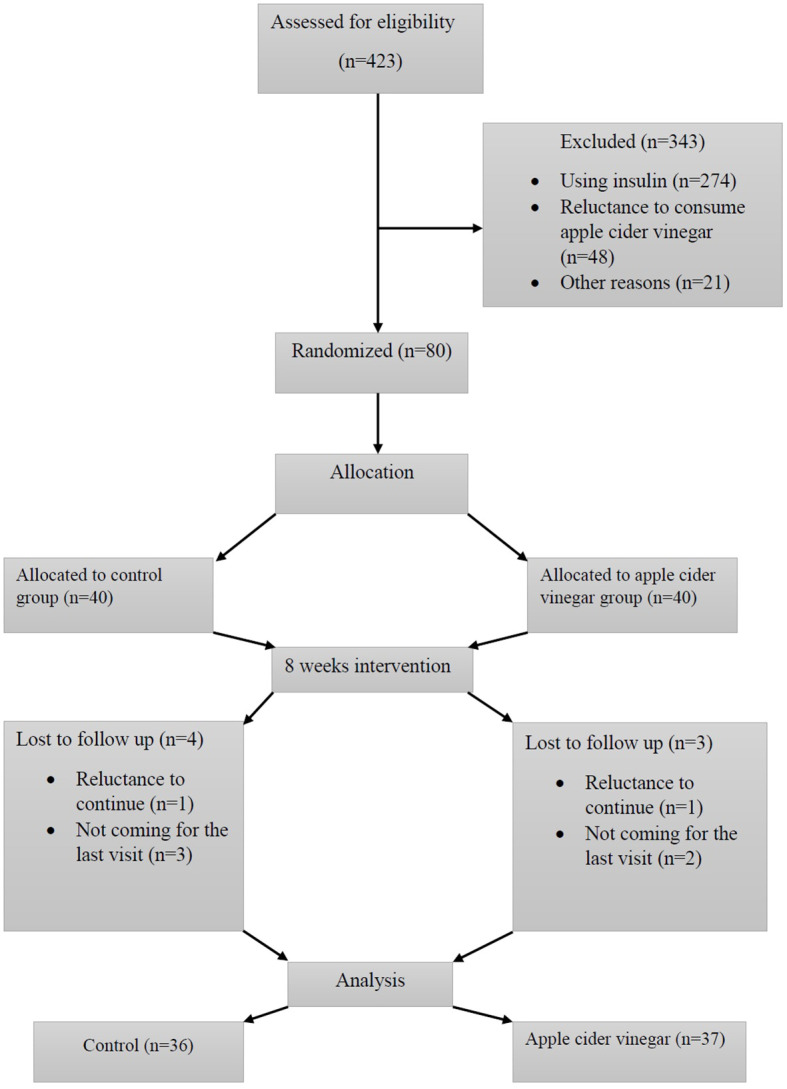
Flowchart of patients’ enrollment and follow-up.

Four hundred and twenty-three diabetic patients were evaluated based on the inclusion and exclusion criteria and 80 patients were enrolled (**Figure 1**).

### 2.4 Randomization

Two of the researchers matched the participants in pairs according to age and sex. Then, each member of the pair was randomly allocated to one of the two groups (ACV or control). The statistical advisor carried out randomization using a random number generator.

### 2.5 Preparation of apple cider vinegar

In this study, we did not use company-manufacturing vinegar, we made it ourselves. A standard protocol was used to produce ACV (22). Briefly, the apples were cut into pieces after washing, and removing the wooden parts and seeds. Then, the apples were poured into clean ten-gallon kegs, and a two-liter cold boiled water was added to each keg. A small amount of salt (20 grams) was added to each keg. The contents of the kegs were stirred daily with a big wooden spoon for 45 days to prepare apple cider vinegar. Then we filtered the vinegar through thin, clean cloth filters. We produced 240 liters of ACV and packed them in one-liter bottles. ACV compounds were examined and approved by the Food Chemistry Laboratory of the School of Allied Medical Sciences (Ahvaz Jundishapur University of Medical Sciences) based on the “Iranian National Standard for Spices and Seasoning - Vinegar - Test Methods 2015”. The acidity, alcohol, pH and Brix of the examined ACV were 5.04%, 0.1%, 2.73 and 5.2°Bx respectively.

### 2.6 Intervention

The duration of the intervention was eight weeks. During the intervention, participants in both groups received the same recommendations for a healthy diet. The recommendation was based on the Iranian healthy food pyramid (23) and included the consumption of healthy carbohydrates (such as fruits, vegetables, whole grains, legumes, fiber-rich foods and low-fat dairy products) and avoiding less healthy foods (foods or drinks with added fats, sugars and sodium). Moreover, it was recommended that the two groups not consume any vinegar or fermented products during the intervention period (which might affect the result of the study). In addition, the treatment group received 30 ml of ACV. It was recommended they consume ACV with lunch or immediately after it. Each month, ACV was given them in one-liter bottles, with a 30 ml graduated cylinder. They could dilute the ACV with 100 ml of water. Patients were followed up weekly by telephone. Compliance was measured by the amount of empty ACV bottles returned every month, which represented a percentage of the ACV consumed during the intervention period.

### 2.7 Expected outcomes of the study

The primary outcomes were fasting blood glucose, insulin, total cholesterol, triglyceride, high-density lipoprotein-cholesterol, low-density lipoprotein-cholesterol, hemoglobin A1C (HbA1C) and homeostatic model assessment for insulin resistance. The secondary outcomes were anthropometric indices and blood pressure. Primary and secondary outcomes were measured before the intervention period and at the end of eight weeks.

### 2.8 Anthropometric assessment

All measurements were performed by a trained technician. The weight of each patient was measured with light clothing and with an accuracy of 100 grams using a weight scale (Seca 760, Germany). The height of each patient was measured with a stadiometer (Seca 217, Germany) in a standing position without shoes and with an accuracy of 0.5 cm while the patient was looking forward. Waist circumference was measured with an inelastic tape, (Seca 201, Germany), with an accuracy of 0.5 cm in standing position, in the area between the last rib and the iliac bone. Hip circumference was also measured with the same tape with an accuracy of 0.5 cm, at the largest circumference of the buttocks. Body mass index (BMI) was determined using the formula (weight in kilograms divided by height squared in meters). Waist-to-hip ratio (WHR) was obtained by dividing the waist by the hip circumference.

### 2.9 Blood pressure measurement

After 15 minutes of rest, systolic and diastolic blood pressures were measured twice with an interval of one minute from the right hand, using a mercury sphygmomanometer (Erkameter 3000, Germany). The mean was reported as blood pressure.

### 2.10 Biochemical measurements

After 12 to 14 hours of fasting, 10 ml of brachial vein blood was taken from patients while sitting on a chair. Blood samples were poured into two separate tubes to isolate serum (without any anticoagulant) and to collect the whole blood (containing EDTA anticoagulant). Tubes without any anticoagulant were centrifuged at room temperature for 15 minutes at 3500 rpm (Hettich centrifuge, ROTOFIX 32 A, Germany). Sera were allocated into two 2.5 ml micro-tubes and were used to measure triglyceride (TG), high-density lipoprotein (HDL), low-density lipoprotein (LDL), fasting blood glucose (FBG), and total cholesterol. They were measured with an autoanalyzer (Miura, Italy) using diagnostic kits (Pars Azmun Co. Iran). Insulin levels were measured using the ELISA kit (monobind, USA) by an ELISA reader (Hiperion, Germany). Whole blood samples were used to measure the HbA1C. The amount of HbA1C was measured with the autoanalyzer (Miura, Italy) using the diagnostic kit (Pishtazteb Co., Iran). Homeostatic Model Assessment for Insulin Resistance (HOMA-IR) was calculated with the following formula:


$$\;\frac{insulin\;\left(\frac{mU}{L}\right)\;\times \;glucose\;\left(\frac{mg}{dl}\right)}{405}$$

### 2.11 Additional questionnaires

In a calm and stress-free environment, the general questionnaire, a 24-hour food recall and the international physical activity questionnaire were completed by asking the participants. The validity and reliability of the physical activity questionnaire have been confirmed in the Iranian population (24). After completing the 24-hour food recall, dietary intake was analyzed using Nutritionist IV software (First Databank, San Bruno, CA, USA).

### 2.12 Statistical analysis

The Kolmogorov-Smirnov test was used to examine the normal distribution of data. The frequency of qualitative data was expressed by the number (percentage). Quantitative data were determined by mean ± standard deviation. To compare quantitative variables between the two groups, an independent *t*-test was used and in cases of non-normal distribution, the Mann-Whitney test was considered. The Chi-square test was used to compare qualitative variables. The paired *t*-test was used to compare the quantitative variables before and after intervention in each group and the Wilcoxon test was used in cases of non-normal distribution. The effect of the co-variant was removed using analysis of covariance (ANCOVA). Analysis was performed with SPSS software (SPSS Inc. released 2008. SPSS Statistics for Windows, Version 17.0. Chicago: SPSS Inc.). The significance level was considered less than 0.05.

## 3 Results

Thirty-seven subjects in the treatment group and thirty-six subjects in the control group completed the study (**Figure 1**). The compliance rate of the ACV group was 91%. We found no adverse effects after consuming ACV. The general characteristics of the participants are shown in **Table 1**. The dietary intake of both groups was not significantly different before the intervention. After the intervention, there was no significant difference in energy (**Table 1**), macro-nutrients (carbohydrate, fat and protein) and micro-nutrients (vitamins A, E, K, C, thiamin, riboflavin, folate, B_12_, and minerals such as Iron, calcium, zinc and magnesium) between the two groups, which showed that the food intake of the two groups was homogeneous and there was no significant difference in food intake (data of micro- and macro-nutrients are not shown in the Table).

**Table 1:** Comparison of general and demographic data between the two groups.

Variables		ACV ^α^ n = (37)	Control ^β^ n = (36)	p-value ^£^
**Age (year)** Mean ± SD		53.11 ± 5.64	52.94 ± 8.46	0.92
**Gender, n (%)**				0.61
	Female	25 (52.1)	23 (47.9)	
	Male	12 (48)	13 (52)	
**Ethnicity, n (%)**				1
	Arab	26 (51)	25 (49)	
	Persian	11 (50)	11 (50)	
**Physical Activity, n (%)**				0.38
	Inactive	29 (53.7)	25 (46.3)	
	Moderate	8 (42.1)	11 (57.9)	
	High	11 (44)	14 (56)	
**Job, n (%)**				0.33
	Retired	5 (50)	5 (50)	
	Housewife	22 (56.4)	17 (43.6)	
	Employee	8 (57.1)	6 (42.9)	
	Self-Employment	2 (20)	8 (80)	
**Education, n (%)**				0.65
	Illiterate	4 (44.4)	5 (55.6)	
	High School	21 (51.2)	20 (48.8)	
	Diploma or college	5 (38.5)	8 (61.5)	
	University education	7 (70)	3 (30)	

^α^Received 30 ml of apple cider vinegar and a recommendation for a healthy diet; ^β^Received only a recommendation for a healthy diet. ^£^Independent sample t-test or Chi-square test for quantitative and qualitative variables respectively.

ACV, apple cider vinegar. SD, standard deviation.

The effect of the intervention on anthropometric indices is presented in **Table 2**. Weight, body mass index, waist circumference and hip circumference decreased after the intervention in both groups, but this decrease was greater in the ACV group. The changes in these anthropometric indices were significant between the two groups after the intervention (p < 0.001, **Table 2**).

**Table 2:** Comparison of anthropometric indices between two groups, before and after the intervention period.

Variables		ACV ^α^ n = 37	Control ^β^ n = 36	p-value ^£^
		Mean ± SD	Mean ± SD	
Weight (kg)				
	Before	83.72 ± 14.61	74.26 ± 13.47	0.05
	After	81.35 ± 14.73	73.96 ± 13.56	0.02
	Change	— 2.37 ± 1.67	— 0.29 ± 1.89	<0.001
	p-value ^€^	<0.001	0.36	
BMI (kg/m^2^)				
	Before	30.70 ± 5.35	28.28 ± 4.84	0.04
	After	29.82 ± 5.35	28.15 ± 4.72	0.16
	Change	— 0.87 ± 0.63	— 0.13 ± 0.76	<0.001
	p-value ^€^	<0.001	0.30	
Waist Circumference (cm)				
	Before	104.29 ± 10.57	99.50 ± 10.32	0.05
	After	101.43 ± 11.13	99.45 ± 10.31	0.43
	Change	— 2.86 ± 2.92	-0.05 ± 2.14	<0.001
	p-value ^€^	<0.001	0.90	
Hip Circumference (cm)				
	Before	108.62 ± 11.65	103.61 ± 7.98	0.03
	After	105 ± 11.58	103.25 ± 7.67	0.45
	Change	— 3.62 ± 2.89	— 0.36 ± 2.54	<0.001
	p-value ^€^	<0.001	0.40	
Waist To Hip Ratio				
	Before	0.96 ± 0.08	0.96 ± 0.06	0.83
	After	0.96 ± 0.08	0.96 ± 0.06	0.88
	Change	0 ± 0.02	0 ± 0.02	0.86
	p-value ^€^	0.42	0.18	
Total Energy Intake (Kcals)				
	Before	1864.27 ± 67.8	1950.33 ± 98.95	0.22
	After	1820.48 ± 96.9	1897.27 ± 80.87	0.45
	Change	— 43.79 ± 78.45	— 53.06 ± 80.67	0.16
	p-value ^€^	0.07	0.05	

^α^Received 30 ml of apple cider vinegar and a recommendation for a healthy diet; ^β^Received only a recommendation for a healthy diet; ^£^Independent sample t-test; ^€^Paired t-test.

ACV, apple cider vinegar; BMI, body mass index. SD, standard deviation.

**Table 3** shows the changes in systolic and diastolic blood pressure after eight weeks of intervention. Systolic blood pressure decreased in both groups after the intervention, which was greater in the ACV group. After removing the effect of BMI before the intervention, changes in systolic blood pressure showed a tendency near to significant between the two groups (p = 0.06). There was no significant difference in diastolic blood pressure between the two groups after the intervention.

**Table 3:** Comparison of blood pressure between the two groups, before and after the intervention period.

Variables		ACV ^α^ n = 37	Control ^β^ n = 36	p-value ^£^
		Mean ± SD	Mean ± SD	
Systolic blood pressure (mmHg)				
	Before	137.02 ± 12.49	132.33 ± 16	0.18
	After	131.35 ± 12.45	130.41 ± 15.30	0.52
	Change	— 5.67 ± 8.59	— 1.91 ± 7.74	0.06
	p-value ^€^	<0.001	0.14	
Diastolic blood pressure (mmHg)				
	Before	80.94 ± 10.66	80.61 ± 10.64	0.15
	After	80.67 ± 9.58	80.13 ± 9.98	0.78
	Change	— 0.27 ± 2.87	— 0.47 ± 2.84	0.83
	p-value ^€^	0.57	0.32	

^α^Received 30 ml of apple cider vinegar and a recommendation for a healthy diet; ^β^Received only a recommendation for a healthy diet; ^£^Analysis of covariance after eliminating body mass index measures before the intervention period; ^€^Paired t-test.

ACV, apple cider vinegar; SD, standard deviation.

The effect of intervention on blood glucose indices is presented in **Table 4**. FBG decreased after intervention in both groups, but the reduction was significant only in the ACV group (p = 0.01). HbA1C decreased in the ACV group more than in the control group and this difference was significant between the two groups (p < 0.001). Insulin increased in the ACV group after the intervention and there was a significant difference between the two groups (p < 0.001). HOMA-IR was increased in the ACV group, but decreased in the control group and showed a significant difference (p = 0.04).

**Table 4:** Comparison of blood glucose indices between the two groups, before and after the intervention period.

Variables		ACV ^α^ n = 37	Control ^β^ n = 36	p-value ^£^
		Mean ± SD	Mean ± SD	
FBG (mg/dl)				
	Before	170.45 ± 82.39	138.91 ± 58.71	0.003
	After	147.59 ± 72.75	136.72 ± 43.27	0.44
	Change	— 22.86 ± 53.38	— 2.19 ± 63.63	0.09
	p-value ^€^	0.01	0.72	
HbA1C (percentage)				
	Before	9.21 ± 2.36	8.04 ± 2.70	0.13
	After	7.79 ± 1.95	7.99 ± 2.34	0.73
	Change	— 1.42 ± 2	-0.05 ± 2.76	<0.001
	p-value ^€^	<0.001	0.89	
Insulin (IU/dl)				
	Before	9.24 ± 10.33	7.83 ± 5.82	<0.001
	After	12.17 ± 11.44	7.98 ± 8.32	<0.001
	Change	2.93 ± 5.70	0.15 ± 3.05	<0.001
	p-value ^€^	<0.001	0.87	
HOMA-IR				
	Before	3.80 ± 4.79	3.66 ± 4.71	0.06
	After	4.57 ± 5.18	3.21 ± 3.76	<0.001
	Change	0.77 ± 3.29	— 0.45 ± 1.51	0.04
	p-value ^€^	0.16	0.08	

^α^Received 30 ml of apple cider vinegar and a recommendation for a healthy diet; ^β^Received only a recommendation for a healthy diet; ^£^Analysis of covariance after eliminating body mass index measures before the intervention period; ^€^Paired t-test.

ACV, apple cider vinegar; FBG, fasting blood glucose; HbA1C, hemoglobin A1C; HOMA-IR, homeostatic model assessment of insulin resistance; SD, standard deviation.

Changes in lipid profile after eight weeks of intervention are presented in **Table 5**. LDL was decreased in the ACV group but increased in the control (p < 0.001). HDL was increased in the ACV and decreased in the control, and after removing the effect of BMI before the intervention, this difference showed a tendency near significant (p = 0.06).There was a significant difference in the changes of total cholesterol, LDL/HDL ratio and cholesterol/HDL ratio after the intervention, between the two groups (p = 0.003, p = 0.001 and p = 0.001, respectively, **Table 5**).

**Table 5:** Comparison of blood lipid levels between the two groups, before and after the intervention period.

Variables		ACV ^α^ n = 37	Control ^β^ n = 36	p-value ^£^
		Mean ± SD	Mean ± SD	
Triglyceride (mg/dl)				
	Before	148.35 ± 64.49	128.55 ± 68.97	0.30
	After	148.10 ± 56.51	128 ± 64.82	0.28
	Change	— 0.24 ± 40.11	— 0.55 ± 45.44	0.77
	p-value ^€^	0.97	0.94	
LDL (mg/dl)				
	Before	110.62 ± 40	104.5 ± 29.74	0.40
	After	85.59 ± 30.20	106.77 ± 38.51	0.03
	Change	— 25.02 ± 26.25	2.27 ± 37.62	<0.001
	p-value ^€^	<0.001	0.71	
HDL (mg/dl)				
	Before	41.10 ± 9.09	45.66 ± 13.86	0.01
	After	42.78 ± 9.27	44.30 ± 10.94	0.07
	Change	1.68 ± 4.35	— 1.36 ± 6.85	0.06
	p-value ^€^	0.01	0.24	
Cholesterol (mg/dl)				
	Before	180.78 ± 46.66	176.02 ± 37.53	0.77
	After	156.78 ± 37	175.25 ± 39.39	0.11
	Change	— 24 ± 30.03	-0.77 ± 39.23	0.003
	p-value ^€^	<0.001	0.89	
LDL/HDL (ratio)				
	Before	2.85 ± 1.30	2.54 ± 1.33	0.21
	After	2.10 ± 0.85	2.52 ± 1.05	0.15
	Change	— 0.75 ± 0.81	— 0.02 ± 0.96	0.001
	p-value ^€^	<0.001	0.90	
Cholesterol/HDL (ratio)				
	Before	4.65 ± 1.72	4.25 ± 1.91	0.27
	After	3.86 ± 1.17	4.22 ± 1.49	0.15
	Change	— 0.78 ± 0.99	— 0.03 ± 1.08	0.001
	p-value ^€^	<0.001	0.84	

^α^received 30 ml apple cider vinegar and recommendation for a healthy diet; ^β^received only recommendation for a healthy diet; ^£^Analysis of covariance after eliminating body mass index measures before the intervention period; ^€^Paired t-test.

ACV, apple cider vinegar; LDL, low density lipoprotein; HDL, high density lipoprotein; SD, standard deviation.

## 4 Discussion

This study was performed to determine the effect of ACV on blood glucose indices and lipid profile in patients with type 2 diabetes and showed positive results. The first positive effect of ACV was the improvement of anthropometric indices. Weight, BMI, waist circumference and hip circumference decreased in subjects who consumed ACV. These results were consistent with the results of Khezri et al. (20). They reported that consuming 30 ml of ACV per day for 12 weeks along with dietary restriction significantly reduced body weight, hip circumference, and abdominal obesity compared with people who had only dietary restriction (20). Kausar et al. showed that consuming 15 ml of ACV for 3 months in patients with type 2 diabetes can lead to a significant reduction in the waist-to-hip ratio (25). Although the waist-to-hip ratio in our study did not significantly decrease after eight weeks of intervention with ACV, it seems that differences in the duration of the intervention could be influential. One possible mechanism of action of vinegar is lipogenesis reduction by suppressing lipogenic genes in the liver and inhibiting transcription factors influencing the conversion of glucose to fat (26). In addition, the effect of vinegar on weight loss has been mentioned through growing lipolysis by increasing the expression of several lipolytic genes (27). In a study, researchers pointed to a decrease in appetite in subjects who consumed ACV, and as a result, the reason for weight loss was a decrease in food intake (20). Another possible mechanism of vinegar is the reduction of energy intake by lowering the glycemic index of foods (28). It seems these two mentioned factors are not the main mechanisms in reducing anthropometric indices in our study, because by examining the dietary intake, no reduction in daily energy intake was observed in the ACV group.

In the present study, although the result did not show a difference between the two groups in blood pressure after the intervention period, a decreasing tendency was observed in systolic blood pressure. It seems that only two studies have examined the effect of ACV on blood pressure. Thinathayalan et al. showed that five days of consumption of ACV by medical students reduced diastolic blood pressure (29). The results of this study are different from our results due to differences in subjects and duration of intervention. Gheflati et al. showed that consumption of 20 ml of ACV for eight weeks in patients with T2D and hyperlipidemia did not affect systolic and diastolic blood pressure (30). To the best of our knowledge, there is no other study about the effect of ACV on blood pressure. We think that the lowering effect of ACV on blood pressure is related to the reduction of anthropometric indices in the present study. Evidences suggest that a decrease in BMI leads to a reduction in blood pressure, especially systolic blood pressure (31–33). Kondo et al. suggested that acetic acid increases calcium absorption, which may trigger the influx of calcium into renin-secreting cells and inhibit renin secretion (34). Therefore, it is possible that foods containing acetic acid (such as ACV), can reduce blood pressure.

The results of our study showed a significant decrease in FBG, HbA1C, total cholesterol, LDL, LDL/HDL ratio, cholesterol/HDL ratio, and an increase in insulin hormone in ACV group. In addition, we observed an increasing tendency in HDL levels. The results of a study in patients with diabetes showed a significant reduction in FBG, HbA1C, and total cholesterol after consuming ACV (25). The results of a study on overweight and obese subjects were in line with the results of our study. It showed that consuming 30 ml of ACV per day for 12 weeks along with dietary restriction significantly reduced total cholesterol, LDL, and increased HDL compared to subjects who had only dietary restriction (20). In another study, consuming two tablespoons of ACV before going to bed at night reduced fasting glucose in the morning (35). The result of this study was similar to our results because we found a decrease in FBG after consuming ACV.

One of the mechanisms of action of ACV is the effect of acetic acid on disaccharidases and inhibition of their activity and thus a reduction in blood glucose (15). Another mechanism is the inhibition of α-amylase and thus a decrease in blood glucose indices (14). In addition, apple cider vinegar reduces the rate of gastric emptying, which causes a reduction in blood glucose (36, 37). It seems the effect of ACV on blood glucose indices is greater than the effect of acetic acid alone (38). Examination of diabetic rats showed that ACV could reduce blood glucose indices by increasing glucose uptake by the liver and muscle, inhibiting glycolysis through glucose-6-phosphate accumulation and increasing glycogen synthase (39–41). An animal study showed that chlorogenic acid (the main polyphenol in ACV) inhibits glucose-6-phosphatase in rats. Inhibition of this enzyme reduces glucose production in both gluconeogenesis and glycogenolysis processes and leads to a decrease in blood glucose (42).

Suggested mechanisms of action of ACV on reducing lipid profile include acetic acid inhibitory effect on enzymes involved in the synthesis of fats, such as ATP citrate-lyase, HMG CoA reductase and fatty acid synthase. Increased excretion of bile and fecal acids was observed in acetic acid-fed rats. It may be one of the mechanisms of action of ACV in the reduction of cholesterol (43). Another mechanism can be expressed by the ACV effect on weight loss. Evidences suggest that weight loss can reduce lipid profile and improve blood glucose indices (44, 45). In this study, a significant decrease in anthropometric indices was observed. Therefore, the reduction of lipid profile can be the result of weight loss and reduction of participants’ BMI.

### 4.1 Main strengths and limitation

One of the limitations of this study is the lack of a placebo. We could not measure the antioxidant indices of ACV, so it is another limitation of the study. The strengths of this study include the appropriate sample size and duration of intervention. Most of the previous studies had an intervention period less than a month; but in this study, the duration of the intervention was eight weeks, which enabled us to investigate the long-term effects of ACV consumption. The other strength of the study was using homemade ACV, which could be made at home and showed its improving effect on controlling diabetes. However, more definitive conclusions about the effectiveness of ACV as a functional food in diabetes management need further research, including larger, longer-term studies with diverse populations.

## 5 Conclusion

Consuming 30 ml of ACV for eight weeks can improve blood glucose indices, anthropometric indices, lipid profile and systolic blood pressure in adult patients with T2D. It seems that consuming ACV as a functional food along with medications can reduce cardiovascular disease risk factors in diabetic patients.

## Data availability statement

The raw data supporting the conclusions of this article will be made available by the authors, without undue reservation.

## Ethics statement

The studies involving humans were approved by the Ethics Committee of Ahvaz Jundishapur University of Medical Sciences/IR.AJUMS.REC.1397.476. The studies were conducted in accordance with the local legislation and institutional requirements. The participants provided their written informed consent to participate in this study.

## Author contributions

SJ: Conceptualization, Supervision, Writing – review & editing. M-RE: Investigation, Writing – original draft. AM: Conceptualization, Writing – review & editing. AK: Methodology, Writing – review & editing. M-HH: Formal Analysis, Writing – review & editing.
